# Mastery of teletherapy is related to better therapeutic relationship and presence in teletherapy: the development of the teletherapy intervention scale

**DOI:** 10.3389/fpsyg.2023.1206960

**Published:** 2023-08-02

**Authors:** Vera Békés, Katie Aafjes-van Doorn, Xiaochen Luo, Sanjeev Balarajan, Christopher J. Hopwood

**Affiliations:** ^1^Ferkauf Graduate School of Psychology, Yeshiva University, Bronx, NY, United States; ^2^Department of Counseling Psychology, Santa Clara University, Santa Clara, CA, United States; ^3^Department of Psychology, University of Zurich, Zurich, Switzerland

**Keywords:** teletherapy, therapeutic presence, therapeutic intervention, working alliance, attitudes

## Abstract

**Introduction:**

Providing teletherapy requires a unique therapeutic approach and mastery of the teletherapy context. We aimed to develop a self-report scale for therapeutic interventions pertinent to teletherapy, and to examine its relationship with teletherapy process variables, and therapists’ attitudes towards teletherapy technology.

**Method:**

A total of 839 therapists participated in a survey study that included standardized measures of therapeutic process (real relationship, working alliance, therapeutic presence), attitudes towards and intention to use teletherapy in the future, and a list of 13 teletherapy intervention items that we hypothesized to be specific to the teletherapy format.

**Results:**

Twelve of the 13 teletherapy intervention items loaded on one factor, with good reliability. The 12-item Teletherapy Intervention Scale was positively related to working alliance, the real relationship, therapeutic presence in teletherapy sessions, as well as to positive attitudes towards teletherapy and intention to use teletherapy in the future.

**Discussion:**

Aspects specific to the practice of teletherapy may be successfully captured by a self-report scale, and adequately navigating the challenges and opportunities of teletherapy might enhance the therapeutic process. Further studies are needed to provide additional validation of the scale, and in how to best use this Teletherapy Intervention Scale in research and clinical training.

## Introduction

Although the use of teletherapy is increasingly common, and the therapeutic outcomes appear to be similar to that of in-person therapy (e.g., Lin et al., 2022), teletherapy comes with unique therapeutic challenges and opportunities (Aafjes-van Doorn et al., 2021; Békés et al., 2021a,b; Aafjes-van Doorn, 2022). Despite the clear benefits for patients who otherwise could not have access to mental health care, therapists have long been reluctant to use teletherapy in their practice. Therapists have expressed concerns about its efficacy and about their own ability to create a strong working alliance via videoconferencing (Brooks et al., 2020; Perry et al., 2020). These concerns greatly impacted attitudes towards teletherapy and hindered its utilization (Connolly et al., 2020). The global transition to teletherapy in 2020 provided an opportunity for therapists and patients to get familiar with teletherapy and obtain first-hand experience of this treatment format. Since then, teletherapy has become part of standard practice for many clinicians (Van Daele et al., 2020; Sheperis and Smith, 2021; Kwok et al., 2022), and a vast amount of research has suggested comparable efficacy with in-person treatment (e.g., Lin et al., 2022). Lots has been written about therapists’ experiences of the therapeutic process in teletherapy during the pandemic (Békés et al., 2020; Perry et al., 2020; Rosen et al., 2020; Van Daele et al., 2020; Hanley and Wyatt, 2021; Helps and Le Coyte Grinney, 2021; Nuttman-Shwartz and Shaul, 2021; Poletti et al., 2021; Machluf et al., 2022; Stukenberg et al., 2022; Aviram and Nadan, 2023). These studies showed that overall, therapists had a reasonably favorable experience, often better than they expected, and their attitudes toward teletherapy became more positive (Békés et al., 2020; Humer et al., 2020). Many therapists were able to do their therapeutic work by making minimal adjustments. However, many other studies also highlighted unique therapeutic challenges, such as a lack of emotional connection with patients, being more easily distracted during sessions, and difficulty maintaining privacy and a professional frame (Aafjes-van Doorn et al., 2021; Békés et al., 2021a,b; Shklarski et al., 2021). Maybe unsurprisingly, therapists also felt less competent in using their therapeutic skills (e.g., warmth) and extra-therapeutic influence (e.g., providing resources), when doing teletherapy (Lin et al., 2021).

### Qualitative findings about teletherapy interventions

The initial quantitative studies were followed by more in-depth qualitative investigations of therapists’ lived experience and found further examples of therapists’ ways of using some new opportunities and overcoming the hurdles of the therapeutic process via videoconferencing. Several therapeutic opportunities were highlighted: First, several qualitative studies reported on a more balanced power dynamic, and the ability to relate in a more genuine human-to-human way. For example, a “democratizing” effect was noted, as patients are now in their own “territory,” instead of entering the therapists’ official space, making the therapy situation feel more equal (Simpson et al., 2019; Mitchell, 2020). Therapists noted that they had become more open and willing to share their personal experiences compared to their in-person practice (Mitchell, 2020). Similarly, some therapists (as well as patients, see Shtrackman, Békés et al., 2023) reported a sense of connecting more as humans besides professional and patient, and letting patients see more of them as persons (Békés et al., 2023). Some therapists reported that they used self-disclosure as a tool to compensate for the physical distance, especially when supporting patients during a time of global distress (Nuttman-Shwartz and Shaul, 2021; Aafjes-Van Doorn et al., 2023). This increased self-disclosure appeared to be related to an increase in self-disclosure of the patient, and might thus indeed have been therapeutic (Luo et al., under review).

Interestingly, although some boundaries were loosened, other boundaries became easier to keep. Among the several advantages noted regarding teletherapy, for example therapists found it easier to start and end the sessions on time in teletherapy than they did in their in-person sessions (Aafjes-van Doorn et al., 2022). As noted by therapists, another opportunity in teletherapy is accessing the patients’ home environment via the screen. Many therapists were also able to take advantage of the opportunity to actively ask and gain more insight into the patients’ home, family, and everyday life.

However, therapists also reported several challenges in teletherapy. For example, therapists noted that the teletherapy sessions often feel less deep emotionally and more superficial, and the teletherapy setting pulls them to provide support and counseling rather than engaging in a more open-ended exploration of the patients’ inner world (Békés et al., 2023). Therapists often attempted to compensate for this by becoming more active and directive, and avoiding silences in teletherapy sessions. In addition, therapists reported that creating emotional closeness via teletherapy required a more active effort (McCoyd et al., 2022). Further, some therapists reported that it was more difficult to read their patients’ emotions and they tended to feel more disconnected from their patients; as one therapist put it: “The one thing I am missing is the feel in the room” (Békés et al., 2023). In order to make up for the lacking nonverbal signals from body movement, therapists tended to make an effort to express their own feelings and emotional reactions verbally. Therapists also noted that it was more challenging to stay focused and present in teletherapy sessions and they get more easily distracted in teletherapy by online activities (e.g., notifications popping up on the screen, zoom fatigue) and offline activities in their home environment (e.g., family members, pets; Békés et al., 2020, 2021a,b; Shklarski et al., 2021; McCoyd et al., 2022).

### Psychotherapy process in teletherapy

In contrast to skeptical expectations, several teletherapy studies conducted during the pandemic suggest that the quality of the therapeutic relationship tends to be of similar, regardless of the in-person or teletherapy format (therapist-reported ratings for a typical teletherapy session and in-person therapy session in survey studies; Aafjes-van Doorn et al., 2021; Békés et al., 2021a,b). Initial studies on the real relationship, another aspect of the therapeutic relationship concerning the genuine, sincere, and realistic perceptions between therapist and patient, indicated that therapists may actually report relatively higher quality of real relationship in their typical teletherapy session than their typical in-person therapy (Aafjes-van Doorn et al., 2020; Békés et al., 2020, 2021a,b).

Besides these aspects of the therapeutic relationship (working alliance and the real relationship), the maintenance of therapeutic presence has also been argued to be a precondition for effective therapeutic relationships and a positive working alliance in teletherapy (Haddouk et al., 2018; Hilty et al., 2019; Geller, 2021; Ruble et al., 2021). Given the many potential technical distractions and concerns about not feeling connected in teletherapy (Aafjes-van Doorn et al., 2020) therapists might find it particularly challenging to achieve therapeutic presence.

Recent teletherapy research conducted at the tail end of the pandemic in 2022 (using the same dataset we use in this study) found that therapists reported feeling significantly less present in teletherapy and their perceptions of the real relationship were somewhat impacted, but there were no average effects on their perceived quality of the working alliance (Aafjes-Van Doorn et al., 2023). Furthermore, other studies found that besides more positive attitudes towards teletherapy in general during the pandemic, therapists who reported stringer therapeutic relationship in teletherapy with their patients, also reported more positive attitudes towards teletherapy and more intention to use it in the future (Békés et al., 2021a,b).

### Aims

The first aim of our study was to develop a therapist self-report scale for the use of therapy interventions pertinent to teletherapy based on findings from previous qualitative studies on patients’ and therapists’ experiences of teletherapy (Aafjes-Van Doorn et al., 2023; Békés et al., 2023). Second, we aimed to explore how this newly developed Teletherapy Intervention Scale relates to the teletherapy process, specifically the therapeutic relationship (therapeutic alliance and real relationship), therapeutic presence, and attitudes towards teletherapy and future intention to use it.

## Methods

### Procedure

The present study includes a subset of data from a larger study comparing in-person and teletherapy processes, which was pre-registered at https://osf.io/qa382/?view_only=ab5158d0656845a6af654937d5b3470e. The present study focuses on hypothesis 12 listed in the pre-registration;^1^ results on other hypotheses based on the same dataset have been published [omitted for peer review]. We collected therapists’ responses on a large-scale survey (see https://osf.io/h9xfz). English speaking licensed therapists and therapists in training were eligible to participate if they had conducted teletherapy via videoconferencing at least once in the past 3 years. Participants were recruited via professional email listservs for clinicians from different mental health professions, therapeutic orientations, and with different patient populations, from graduate programs in counseling, clinical psychology and social work, as well as through professional networks. In addition, information about the study was posted on international social media groups for mental health professionals worldwide (Facebook, Reddit). After signing the therapist consent form, therapists completed an about 20-min anonymous survey. The survey included demographic questions, individual items, and standardized psychotherapy process measures. Participants did not receive any compensation for completing the survey. All study data were collected between March 08, 2022 and June 30, 2022, a period of time during which the COVID-19 incidence rate was relatively low and the social restrictions and mask requirements had been lifted in most countries. The study was approved by the [local - omitted for peer review] institutional review board.

### Participants

A total of 839 therapists of the 1,298 who started the survey completed the Teletherapy Intervention items in the survey (see description below) and were included in the present study. This subsample differed from therapists who only started the survey but did not complete the Teletherapy Intervention items: Completers were older, *t* (1296) = 2.30, *p* = 0.003, with more clinical experience, *t* (1271) = 3.31, *p* < 0.001 more process-oriented (rather than cognitive-behavior) in their primary therapy approach, *t* (988) = 5.42, *p* < 0.001. There was no difference based on reported gender *t* (1283) = 0.63, *p* = 0.529, or licensure *t* (1296) = 0.15, *p* = 0.88. The average age of the participating therapists was 42.87 years old (SD = 16.60). Most therapists identified as female (*n* = 549; 65.4%), White (*n* = 570, 67.9%), and North American (*n* = 742; 88.5%). Most of the therapists were trained in psychology (*n* = 436; 52%) or social work (*n* = 108; 12.9%) with an average of 10.76 (SD = 6.89) years of clinical experience and 15.86 sessions (SD = 10.51) per week. Most therapists identified with the Psychodynamic (*n* = 242; 28.8%) or Psychoanalytic (*n* = 180; 21.5%) approach, and treated adults (*n* = 585; 69.7%) or adolescent patients (*n* = 122; 14.5%). Detailed demographic data about the study sample is presented in Supplement A.

### Measures

The individual items and standardized measures used in this survey can be found at https://osf.io/qa382/?view_only=ab5158d0656845a6af654937d5b3470e. The instruction of the standardized measures was adapted to ask participants to respond considering their “typical experience” in teletherapy [adapted from Lin et al. (2021) and Probst et al. (2021)].

#### Teletherapy intervention scale

We included 13 new items that reflect therapists’ mastery of the teletherapy setting, that is, their use of the opportunities and counteracting the inherent challenges specific to the teletherapy setting. The items were developed based on a review of previous qualitative studies on therapists’ experiences regarding the specifics of the teletherapy process and interventions. Authors of previous qualitative studies on teletherapy acted as experts in reviewing and editing these items so that they capture the essence of therapists reported experience [omitted for peer review]. Items aimed to capture ways that therapists cope with and counteract certain challenges posed by teletherapy (e.g., being active in sessions to compensate for a sense of disconnection, verbalize feelings to compensate for reduced nonverbal cues, being more humane as opposed to professional to facilitate a sense of closeness despite physical distance), other items are related to positive experiences despite the challenges (e.g., managing to feel focused in session and attuned to the patients despite commonly experienced challenges with these, deepening the sessions despite a pull to stay on a more superficial level), while other items described taking advantage of opportunities arising through the tele-sessions (e.g., exploring the patients’ home environment, starting and ending sessions on time).

Specifically, the 13 items were the following, based on a 1–5 Likert rating scale, ranging from 1- Not at all typical, to 5 - Very typical: (1) I share my personal experiences with my patients; (2) I am emotionally attuned to my patients; (3) I am active in session, trying to engage the patient and direct the session; (4) I express my feelings not only in my face/tone, but I also verbalize my feelings explicitly; (5) To understand my patient’s feelings I rely on nonverbal signals; (6) I let patients see me as I really am; (7) I am fully focused and present in the sessions; (8) The sessions are deep, intense (as opposed to superficial); (9) We connect as humans besides professional and patient; (10) I tend to start and end my sessions on time; (11) I am comfortable with the use of silences in my sessions; (12) I make active efforts to connect emotionally with my patient; (13) I express curiosity about the patients’ home environment.

In this study, the Chronbach’s alpha of this scale was 0.84.

#### Working Alliance

Therapeutic alliance was assessed with the Working Alliance Inventory - Short Revised - Therapist (WAI-SRT; Hatcher and Gillaspy, 2006). The WAI-SRT is a 10-item scale that uses a five-point Likert scale, ranging from *seldom* (1) to *always* (5). Following Bordin’s (1979) theoretical model, the WAI-SRT has three subscales: Bond, Goal, and Task. Cronbach’s α for teletherapy WAI-SRT was 0.89.

#### Real Relationship

The Real Relationship Inventory Therapist Form (RRI-T; Gelso et al., 2005) was used to assess the real relationship. It includes scales measuring realism and genuineness. The RRI-T has altogether 24 items to rate on a 5-point Likert scale from *Strongly Disagree* (1) to *Strongly Agree* (5). Cronbach’s α for the RRI-T overall score was 0.87, for the subscales realism and genuineness were 0.73 and 0.76, respectively.

#### Therapeutic Presence

The Therapeutic Presence Inventory Therapist (teletherapy-T; Geller et al., 2010) is a 21-item self-report questionnaire regarding the therapist’s in-session experience with various aspects of therapeutic presence, including physical, emotional, cognitive, relational, and spiritual aspects. Participants respond on a 7-point Likert sale, ranging from *Not at all* (1) to *Completely* (7). Cronbach’s alpha was 0.80 for this scale in our sample.

#### Attitudes towards teletherapy technology

The Unified Theory of Acceptance and Use of Technology Therapist Version (UTAUT-T; Békés et al., 2022) was used to assess attitudes towards teletherapy. The 21-item UTAUT-T Attitudes subscale includes items related to performance expectancy, effort expectancy, social influence, and facilitating conditions regarding using teletherapy. Additional two items assess behavioral intention, that is, declared intent and plan to use teletherapy in the future. Items of the UTAUT-T scales are scored on a Likert scale ranging from 1 (Strongly disagree) to 5 (Strongly agree). The UTAUT-T has strong psychometric properties (Békés et al., 2023). In the present study, the UTAUT-T’s Cronbach’s alpha was 0.79.

### Data analysis

First, to identify the latent constructs associated with the ratings on the 13 teletherapy intervention items, we conducted exploratory factor analyses (EFAs). EFA is recommended when identifying the factor structure of a newly developed measure with limited evidence to specify a prior factor model (Fabrigar et al., 1999). We used the Maximum likelihood (ML) method because there was no evidence of severe non-normality in the distributions of measured variables. We used the Promax with Kaiser normalization rotation method, which allows the items to be correlated. Two criteria were used to determine the number of factors retained; (1) Assessing rating scores of the 13 items, such that factors with eigenvalues above one were retained; (2) Inspecting a scree plot of the observed eigenvalues ordered from largest to smallest, looking for natural break or drop-off point where the curve flattens off, and using the number of data points above the drop-off point as an indicator of number of factors to retain.

Second, we calculated Cronbach’s alphas to assess the internal consistency of the scale. Third, to establish relationships between teletherapy interventions and other variables, first we used zero-order Pearson correlations and independent samples t-tests to establish whether the Teletherapy Intervention Scale was related to demographic variables, such as age, gender, and self-reported primary therapeutic orientation, subsequently, we controlled for significant variables when running Pearson correlational analyses between Teletherapy Intervention Scale and therapeutic alliance, real relationship, therapeutic presence, and attitudes towards teletherapy and intention to use teletherapy in the future variables.

We created a binary variable for self-reported primary therapeutic orientation, which included cognitive and/or behavioral (CBT) approaches versus process-oriented approaches (including humanistic, psychodynamic/analytic, and systemic). Gender was treated as a binary variable (1 = female, 2 = male). The small number of nonbinary participants (*n* = 9) were removed for this covariate analysis.

All statistical analyses were conducted using IBM SPSS Statistics (Version 28).

## Results

### Teletherapy intervention scale

**Figure 1:**
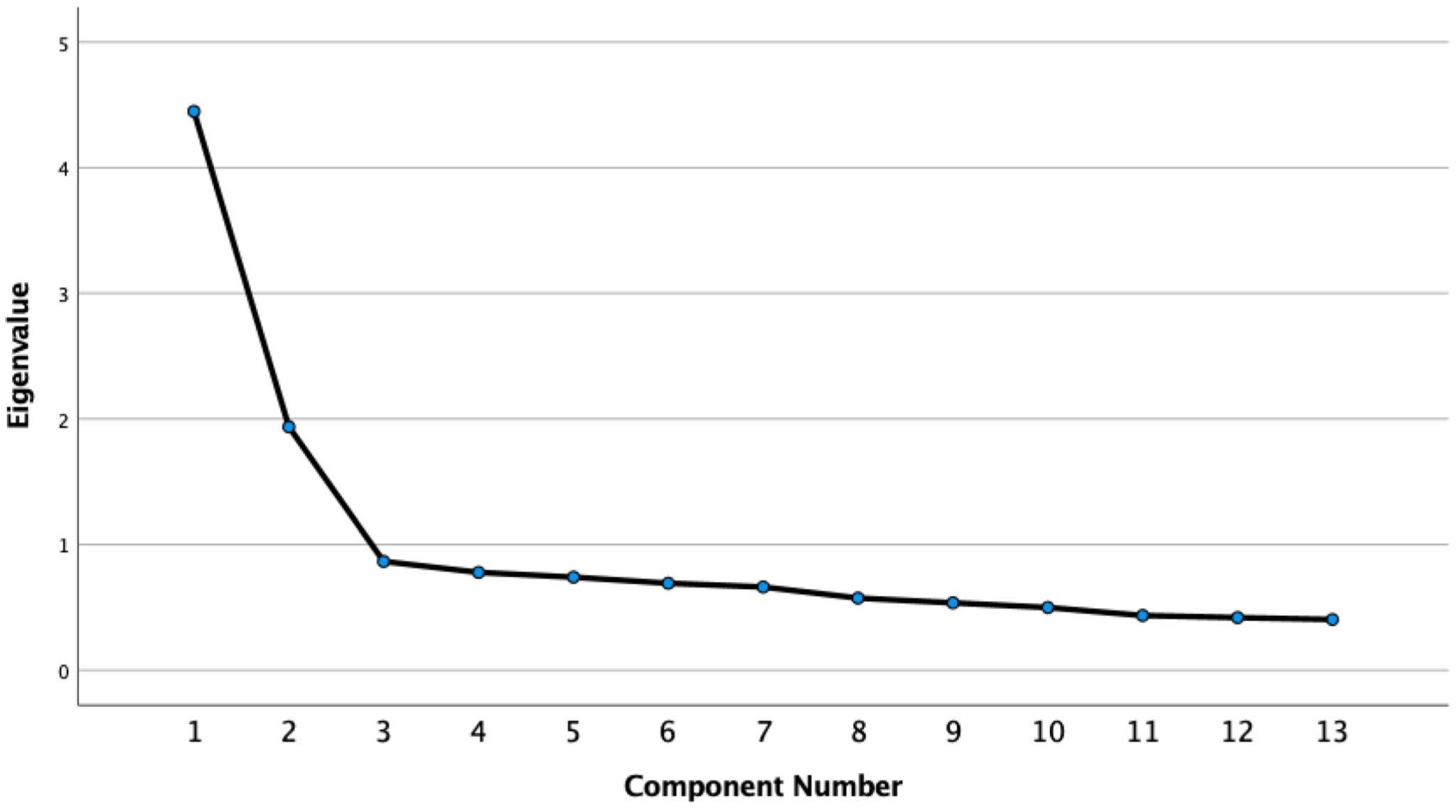
Scree Plot of the 13 Teletherapy Intervention Items in the Exploratory Factor Analysis. The 12-item scale’s Chronbach’s alpha was 0.83, indicating a good internal consistency. Inter-scale correlation coefficients were all *r* > 0.30, *p* < 0.001.

Exploratory factor analysis of the 13 teletherapy intervention items showed a two-factor solution, see Figure 1. However, only one item loaded on the second factor (“*I share my personal experiences with my patients”*), and three items were cross loading on both factors with higher loadings on the first factor. The 13-item items’ factor loadings are included in Table 1. Next, we conducted a reliability analysis for the 13-item scale, which showed that the one item loading on the second factor had weak correlation with the total scale with a correlation coefficient (*r* = 0.291), below the commonly used threshold of *r* < 0.3 (Field, 2013). Therefore, we decided to remove this item from the scale and continue with a 12-item one factor solution. We calculated internal consistency of the scale and correlations with other study variables using this 12-item scale.

**Table 1:** Factor loadings of the teletherapy intervention items.

		Component	
Teletherapy intervention item	Mean (SD)	1	2
1. I share my personal experiences with my patients.	2.69 (1.37)	0.320	0.726
2. I am emotionally attuned to my patients.	4.11 (0.97)	0.640	−0.439
3. I am active in session, trying to engage the client and direct the session.	3.52 (1.25)	0.542	0.401
4. I express my feelings not only in my face/tone, but I also verbalize my feelings explicitly.	3.4 (1.24)	0.578	0.496
5.To understand my patient’s feelings, I rely on nonverbal signals.	3.79 (1.03)	0.622	−0.128
6. I let patients see me as I really am.	3.47 (1.12)	0.548	0.515
7. I am fully focused and present in the sessions.	3.99(0.96)	0.675	−0.304
8. The sessions are deep, intense (as opposed to superficial).	3.87 (0.97)	0.768	−0.337
9. We connect as humans besides professional and patient.	3.71 (1.10)	0.655	0.175
10. I tend to start and end my sessions on time.	4.03 (1.04)	0.454	−0.383
11. I am comfortable with the use of silences in my sessions	3.76 (1.11)	0.595	−0.311
12. I make active efforts to connect emotionally with my patient.	4.04 (1.04)	0.698	−0.188
13. I express curiosity about the patients’ home environment	3.74 (1.17)	0.496	0.144

### The teletherapy intervention scale and other variables

Next, we explored whether the created 12-item Teletherapy Intervention Scale was related to other therapeutic variables. First, we found that Teletherapy Intervention Scale was positively associated with clinical experience (*r* = 0.10, *p* = 0.006), but not with primary therapeutic orientation (CBT or process orientation, *t* (636) = −0.11, *p* = 0.912); therefore clinical experience was controlled for in the subsequent analyses. The Teletherapy Intervention Scale was positively related to the following scales completed for teletherapy: therapeutic alliance, real relationship, and therapeutic presence, as well as attitudes towards teletherapy and behavior intention to use teletherapy in the future, see Table 2.

**Table 2:** Partial correlation between study variables.

Measures	M (SD)	1	2	3	4	5	6
1.Teletherapy Int	3.79 (0.65)	–	–	–	–	–	–
2. WAI	3.98 (0.71)	0.770	–	–	–	–	–
3. RR	3.58 (0.54)	0.518	0.618	–	–	–	–
4. TP	4.85 (0.84)	0.532	0.594	0.696	–	–	–
5. UTAUT-T	3.58 (0.57)	0.472	0.542	0.492	0.561	–	–
6. Beh Intention	3.84 (0.97)	0.340	0.418	0.359	0.411	0.727	–

All correlations are *p* < 0.001. Teletherapy Int, Teletherapy Intervention Scale, WAI, Working Alliance Inventory; RR, Real Relationship; TP, Therapeutic Presence; UTAUT-T, Teletherapy Technology Acceptance for Therapists; Beh, Behavioral.

## Discussion

In this study we aimed to develop a self-report scale to assess interventions specific to teletherapy. This new Teletherapy Intervention Scale intends to assess mastery of teletherapy, that is, coping with and counteracting challenges and using opportunities inherent in the teletherapy setting. We also examined the relationship between the Teletherapy Intervention Scale and other process variables in teletherapy regarding the therapeutic relationship, therapeutic presence, and therapists’ attitudes towards teletherapy technology and intention to use it in the future.

Exploratory factor analysis showed that 12 items out of the originally included 13 items of the Teletherapy Intervention Scale could be conceptualized as representing one underlying construct. One item, (“I share my personal experiences with my patients”), did not load on this factor, possibly because it was not seen as therapeutic *per se*, or reflects a therapeutic stance more generally, rather than a unique teletherapy experience.

The Teletherapy Intervention Scale was positively related to the therapeutic alliance, the real relationship, therapeutic presence in teletherapy, which provides preliminary support for the Teletherapy Intervention Scale’s validity, since it implies that using teletherapy interventions may results in being able to create a better therapeutic relationship with patients and being more present in teletherapy sessions.

Moreover, therapists with higher teletherapy intervention scores also tended to have more positive attitudes towards teletherapy, and they were also more likely to intend to continue using teletherapy in the future. In line with previous research on attitudes towards teletherapy, it is likely that therapists who sufficiently adapt their therapy process to make use of the benefits of teletherapy and address the challenges of therapy, are also more favorable towards teletherapy technology more generally. Previous research suggests that more experience with teletherapy decreases therapists concerns about teletherapy, increases their sense of competency using teletherapy, and also relates to higher perceived therapeutic relationship quality, even in the midst of the pandemic (Békés et al., 2021a,b, 2023; Aafjes-van Doorn et al., 2023).

### Clinical implications

Pending on further future validation of the Teletherapy Intervention Scale, it may have several clinical implications for therapist training. This scale may be used in future process research on teletherapy treatments, as an add-on scale to the validated multitheoretical list of therapeutic interventions (MULTI; McCarthy and Barber, 2009; Solomonov et al., 2019) that was developed for in-person treatments. This might be especially relevant because interventions specific to teletherapy appear to be linked with the working alliance in teletherapy, just as therapy interventions were linked to the quality of alliance following alliance ruptures in in-person therapy (Chen et al., 2020). Of note, the items of the Teletherapy Intervention Scale are transtheoretical, and thus could potentially capture common interventions in various therapeutic orientations. Accordingly, there might also be a bidirectional relation between use of these common teletherapy related factors and the development of the working alliance, as there was for “common factors” techniques and alliance in in-person therapy (Solomonov et al., 2018).

Importantly, conceptually, the better use of teletherapy specific interventions by the therapists may relate to better therapeutic outcomes as well. There is strong evidence for the relationship between the therapeutic relationship and symptom improvement both in teletherapy (Norwood et al., 2018) and in in-person therapy (Cataldo et al., 2021; Smith et al., 2022), and given the relationship between teletherapy interventions and relational variables in our study, teletherapy interventions might also relate to better outcomes in teletherapy.

Moreover, the newly developed Teletherapy Intervention Scale could also be used by graduate schools and training institutes to aid the development of skills in teletherapy. It could, for example, be used as an observer-rated competency scale when evaluation video recorded teletherapy sessions, to assess how therapists in training navigate the unique aspects of the teletherapy process. It could also be used as a self-report scale for therapists themselves when they review their own work and want to identify micro skills they need to target in their deliberate practice. This scale could also be used more generally as a concrete tool to teach therapists about research findings on the teletherapy process and how it might impact their own clinical practice.

### Limitations and future directions

Several limitations and future directions can be identified. First, this study reported on the initial development and validation of a Teletherapy Intervention Scale, and as such it needs further validation. It is surprising that 3 years after the start of the sudden transition to teletherapy, no therapy intervention scale has been developed that taps into the teletherapy context specifically. Therefore, this initial development of the teletherapy intervention scale is important, and needs validation in larger, more diverse samples. Relatedly, a further limitation is that the validity of the standardized scales of working alliance, real relationship and therapeutic presence could be questioned, given that these measures were used to assess the therapists’ experiences with their typical in-person sessions and teletherapy sessions, rather than a specific session with a specific patient as originally intended by the standardized scales.

Second, our study reported on therapists’ perspectives of the frequency of used interventions. We know from previous research that therapists might not be the best judge of what interventions they actually use in their therapy sessions. Further studies are needed to explore differences in therapeutic interventions in in-person and teletherapy settings as perceived not only by therapists but also by patients, and to provide practical guidelines for training and clinical practice in using teletherapy interventions.

Third, this cross-sectional survey study did not report on actual session-by-session ratings of the relational variables, but ratings across typical teletherapy sessions. A longitudinal study investigating session-by-session ratings of these teletherapy interventions would be a welcome validation study for these identified 12 items. Specifically, given that using teletherapy appears to lead to more positive attitudes toward it, it is possible that therapists may also be able to use the teletherapy interventions in better ways; or might feel more comfortable with the use of teletherapy specific interventions when it is no longer associated with the stressful pandemic time (Messina and Loffler-Stastka, 2021). Furthermore, our study did not include treatment outcomes; future studies should assess the potential relationship between the use of teletherapy interventions and treatment efficacy.

### Conclusion

This study is unique in that it operationalizes how exactly therapeutic interventions in teletherapy are different from interventions used in in-person therapy. It reports on the development of a scale for teletherapy interventions which captures therapists’ mastery over the inherent challenges and opportunities of teletherapy, and which could be used for research, professional development, and training purposes. Overall, our findings indicate that certain interventions in teletherapy sessions appear unique to teletherapy and that therapists using these may also be able to experience better relational quality in their teletherapy sessions, be more present in their teletherapy sessions, and had more positive views of and intention to continue using teletherapy.

## Data availability statement

The raw data supporting the conclusions of this article will be made available by the authors, without undue reservation.

## Ethics statement

The studies involving human participants were reviewed and approved by Western IRB, Yeshiva University’s IRB. The patients/participants provided their written informed consent to participate in this study.

## Author contributions

VB developed the concept, collected data, conducted the data analysis, and wrote up the first draft of the manuscript. KA-vD developed the concept, collected data, and wrote the manuscript. XL collected data and edited the manuscript. SB edited the manuscript. CH developed the concept, collected data, and reviewed the manuscript. All authors contributed to the article and approved the submitted version.

## Conflict of interest

The authors declare that the research was conducted in the absence of any commercial or financial relationships that could be construed as a potential conflict of interest.

## Publisher’s note

All claims expressed in this article are solely those of the authors and do not necessarily represent those of their affiliated organizations, or those of the publisher, the editors and the reviewers. Any product that may be evaluated in this article, or claim that may be made by its manufacturer, is not guaranteed or endorsed by the publisher.

## Footnotes

^1^ https://osf.io/96yr7
