# Supplementary material for: Mastery of teletherapy is related to better therapeutic relationship and presence in teletherapy: the development of the teletherapy intervention scale
Source: Front Psychol. 2023 Aug 2;14:1206960. doi: 10.3389/fpsyg.2023.1206960 (PMC10433166; doi:10.3389/fpsyg.2023.1206960)
Supplement: Supplementary file 1 [file Data_Sheet_1.PDF]

Supplement A

Descriptive Statistics (N = 839)

	n	%
What is your gender?		
Female	549	65.4
Male	281	33.5
Non-binary	9	1.1
In which country do you currently reside?		
United States of America	717	85.5
European countries	44	5.3
Canada	25	3.0
Other	53	6.2
*What is your race or ethnicity?***	570	67.9
White	46	5.5
Hispanic or Latino	62	7.4
Asian or Asian Indian	8	1.0
Middle Eastern	110	13.1
American Indian or Alaska Native	38	4.5
African American	22	2.6
Native Hawaiian or Pacific Islander		
*What is your professional field?**		
Social work	108	12.9
Psychology	436	52.0
Counseling	93	11.1
Nursing	37	4.4
Medicine	41	4.9
Marriage and family therapy	49	5.8
Other (e.g., psychoanalyst/psychotherapist)	38	4.5

What is your primary theoretical orientation?		
Behavioral	64	7.6
Cognitive	86	10.3
Humanistic	39	4.6
Psychodynamic	242	28.8
Psychoanalytic	180	21.5
Integrative	144	17.2
Systemic	38	4.5
What patient population do you work with most often?		
Children	57	6.8
Adolescents	122	14.5
Adults	585	69.7
Older adults	37	4.4
Other	38	4.5

Note. * Multiple responses were allowed.
